# Developing a fall prevention intervention economic model

**DOI:** 10.1371/journal.pone.0280572

**Published:** 2023-01-27

**Authors:** Hailey Saunders, Carol Anderson, Fabio Feldman, Jayna Holroyd-Leduc, Ravi Jain, Barbara Liu, Susan Macaulay, Sharon Marr, James Silvius, Jennifer Weldon, Ahmed M. Bayoumi, Sharon E. Straus, Andrea C. Tricco, Wanrudee Isaranuwatchai

**Affiliations:** 1 Knowledge Translation Program, Li Ka Shing Knowledge Institute, St. Michael’s Hospital-Unity Health Toronto, Toronto, Ontario, Canada; 2 Alberta Health Services, Edmonton, Alberta, Canada; 3 Department of Biomedical Physiology and Kinesiology, Simon Fraser University, Burnaby, British Columbia, Canada; 4 Department of Medicine, University of Calgary, Calgary, Alberta, Canada; 5 Ontario Osteoporosis Strategy, Osteoporosis Canada, Toronto, Ontario, Canada; 6 Geriatric Medicine, Sunnybrook Health Sciences Centre, Toronto, Ontario, Canada; 7 Department of Medicine, University of Toronto, Toronto, Ontario, Canada; 8 SPOR Evidence Alliance Project; 9 Cumming School of Medicine, University of Calgary, Calgary, Alberta, Canada; 10 MAP Centre for Urban Health Solutions, Li Ka Shing Knowledge Institute, St. Michael’s Hospital, Unity Health Toronto, Toronto, Ontario, Canada; 11 Division of General Internal Medicine, St. Michael’s Hospital, Toronto, Ontario, Canada; 12 Institute of Health Policy, Management and Evaluation, University of Toronto, Health Sciences Building, Toronto, Ontario, Canada; 13 Epidemiology Division, Dalla Lana School of Public Health, University of Toronto, Toronto, Ontario, Canada; 14 Health Intervention and Technology Assessment Program, Ministry of Public Health, Bangkok, Thailand; University of British Columbia, CANADA

## Abstract

**Purpose:**

Model-based economic evaluations require conceptualization of the model structure. Our objectives were to identify important health states, events, and patient attributes to be included in a model-based cost-effectiveness analysis of fall prevention interventions, to develop a model structure to examine cost-effectiveness of fall prevention interventions, and to assess the face validity of the model structure.

**Methods:**

An expert panel comprising clinicians, health service researchers, health economists, a patient partner, and policy makers completed two rounds of online surveys to gain consensus on health states, events, and patient attributes important for fall prevention interventions. The surveys were informed by a literature search on fall prevention interventions for older adults (≥65 years) including economic evaluations and clinical practice guidelines. The results of the Delphi surveys and subsequent discussions can support the face validity of a state-transition model for an economic evaluation of fall prevention interventions.

**Results:**

In total, 11 experts rated 24 health states/events and 41 patient attributes. Consensus was achieved on 14 health states/events and 26 patient characteristics. The proposed model structure incorporated 12 of the 14 selected health states/events. Panelists confirmed the face validity of the model structure during teleconferences.

**Conclusions:**

There is a dearth of studies presenting the model conceptualization process; consequently, this study involving multiple end user partners with opportunities for input at several stages adds to the literature as another case study. This process is an example of how a fall prevention economic model was developed using a modified Delphi process and assessed for face validity.

## Introduction

Falls are the most common cause of injury hospitalizations in American and Canadian older adults (≥ 65 years) [1, 2]. Fall prevention interventions can decrease these events. A network meta-analysis (NMA) published in 2017 identified seven effective fall prevention interventions: exercise; combined exercise and vision assessment; combined exercise, environmental assessment, and vision assessment; combined environmental assessment and vision assessment; combined environmental assessment and exercise; combined exercise, electromagnetic field therapy and whole body vibration, and calcium and vitamin D supplementation; and combined multifactorial assessment and patient-level quality improvement strategies [3]. Existing cost-effectiveness analyses comparing fall prevention interventions among each other or to no intervention have not examined the relative economic value of the aforementioned interventions [4–16].

One of several methods to assess the cost-effectiveness of fall prevention interventions is decision analytic modeling which allows the comparison of multiple interventions and extrapolation of trial data [17]. Although many decision analytic models for fall prevention interventions exist and there are guidelines for best practices in modeling falls, few modelers provide details on the choice of model structure and the associated assumptions [18]. Without information on model assumptions and their justifications, we could not properly assess whether an existing model is appropriate for our decision problem and instead developed a de novo model.

Guidelines on model conceptualization recommend a transparent and explicit process that involves different end user partners, including health economic modelers and health professionals [19, 20]. Few economic evaluations were found to have documented the model conceptualization process and none were specific to falls [21–24].

Therefore, this study had three objectives:

1. to identify important health states events, and patient attributes for fall prevention interventions;
2. to propose a model structure based on the outcomes of objective 1 to be used in an economic evaluation of fall prevention interventions; and
3. to assess the face validity of the model structure.

## Methods and analysis

In accordance with health economics guidelines and following the process outlined by Afzali and colleagues in their 2019 publication on the conceptualization of a frailty model [21], we formed a planning committee comprising four individuals with expertise in economic evaluations and knowledge synthesis [19, 20]. Our work was conducted in three stages: 1) a literature review on economic evaluations of fall prevention interventions and clinical practice guidelines for falls to inform survey items; 2) a modified Delphi process consisting of two online surveys to obtain high agreement on model states, events, and patient attributes to inform the model structure; and 3) virtual teleconferences to obtain feedback on the overall face validity and assumptions imposed by the model structure.

### Literature review

We conducted literature reviews on economic evaluations of fall prevention interventions and risk factors for falls in older adults to inform the initial list of health states, events, and patient attributes to be used in the modified Delphi process.

#### Economic evaluations

The economic evaluation search was conducted by an information specialist using Ovid Medline, Embase, Cochrane Central Register of Controlled Trials, Cochrane Database of Systematic Reviews, Database of Abstracts of Reviews of Effects, Cochrane Methodology Register, Health Technology Assessment, National Health Service Economic Evaluation Database, and AgeLine, from inception to May 13, 2019. The search strategies, adapted for each database, used a comprehensive combination of subject headings and keywords for fall prevention, combined with a search filter developed by the Centre for Reviews and Dissemination National Health Service Economic Evaluation Database to capture economic evaluations (S1 File). The search was limited to the English or Thai languages. The literature search results were screened for model-based economic evaluations on fall prevention interventions and the model type and health states were summarized for each included study.

#### Clinical Practice Guidelines

We also reviewed the literature on fall risk factors by searching for Clinical Practice Guidelines on preventing falls in older adults. Clinical Practice Guidelines were selected as an appropriate source for risk factors as they summarized key risk factors for screening according to setting. The search was conducted in Ovid Medline from inception to October 23, 2019 using the search strategy for falls from the economic evaluation search, clinical guidelines search terms published by the Canadian Agency for Drugs and Technologies in Health [25] and limited to English language publications (S2 File). Included studies were reviewed for information on risk factors.

### Delphi process

We conducted a modified Delphi process to obtain input on model conceptualization from a wide range of end user partners. The Delphi process, used by three previously identified economic evaluation model conceptualization studies [21, 22, 24], is a method of consensus-building that typically consists of an iterative process of obtaining feedback from a panel of experts. We used a modified online Delphi process in which the initial survey was developed using the literature review rather than an open-ended questionnaire [26].

The aim of the Delphi process was to gain high agreement on the set of health states, events, and patient attributes to include in our model. An individual-level, state transition (microsimulation) model was selected for our analysis, as it allows us to introduce heterogeneity and patient history into our model [27].

Health states are conditions a person can be in and are required to be mutually exclusive and exhaustive [27]. To help respondents identify health states for fall prevention interventions, we asked them to consider whether a health state would have an impact on costs, health-related quality of life, mortality, and progression to other states. Events are experiences that also impact costs, health-related quality of life, mortality, and progression to other states but are distinguished from health states by their temporary nature. Because some conditions and experiences could be considered either a health state or an event, particularly depending on the time horizon of the model, we asked about these items together, including the option to provide input on whether the item should be considered a health state or event.

We used the term “patient attributes” to refer to heterogeneous patient characteristics which may be associated with the risk of a fall, risk of injury after a fall, type of injury after a fall, cost of treating an injury after a fall, disease progression, clinical pathway after a fall (e.g., treatment, hospitalization, rehabilitation hospitalization, admittance to long-term care), quality of life, resource use, or mortality.

#### Panel members

Experts for the Delphi panel were recruited from the project grant team and invited via email in October 2019. The project grant team comprised 19 individuals with experience in economic evaluations, decision modeling, knowledge synthesis, applied geriatric research, fall prevention, and health policy decision-making representing Alberta (n = 4), British Columbia (n = 1), and Ontario (n = 14). We aimed to have the maximum number of panel members within the constraint of our recruitment pool. Panel members provided written consent to participate in the modified Delphi process via email and surveys were administered using SurveyMonkey, a cloud-based software that supports online survey development and distribution [28].

#### Surveys

Surveys were developed in SurveyMonkey [28] by the planning committee, guided by the format used by Afzali and colleagues and using the literature review results [21, 28]. The first survey was pilot tested with four people recruited from research teams at our institution and not included on the panel. Pilot testers provided feedback on the questions and suggested rewording. The first survey was circulated on February 10, 2020 with a deadline of February 24, 2020. The second survey was circulated on March 30, 2020 with a deadline of April 27, 2020. All definitions for high agreement, criteria to drop items and stopping criteria were determined a priori, using the definition of high agreement established by Schneider and colleagues (Table 1) [29].

**Table 1:** Criteria for Delphi methods adapted from Schneider and colleagues [29].

**Definition of high agreement**
♦ Inclusion: ≥ 80% of respondents rate an item four or greater on a scale from zero to five
♦ Exclusion: ≥ 80% of respondents rate an item two or less on a scale from zero to five
♦ Non-consensus: All other possibilities
**Criteria to drop items**
♦ Excluded items based on above criteria will not move forward to next round.
**Stopping criteria**
♦ The modified Delphi process will end when high agreement is reached or after three rounds of surveys, whichever comes first. If high agreement is not reached, the planning committee will make a final determination for items for which high agreement was not reached based on survey responses and by reaching out to individuals with contrasting scores for discussion.

An introduction to the survey presented concepts of economic evaluations such as state transition models, time horizon and cycle length. The first survey consisted of eight questions and a conflict of interest declaration (S3 File). In the first question, panel members were asked to rate potential health states/events on a Likert scale from 0 to 5 representing no impact to very strong impact. Panel members rated patient attributes based on the strength of their potential association with falls from 0 to 5 representing no association to very strong association. A “Don’t Know” option was given to dissuade non-informative responses. There was an opportunity for respondents to suggest additional health states, events, and patient attributes.

The second survey was personalized for each panel member, such that their rating and the group mean ratings from the first survey were indicated for each item (S4 File). Items that reached high-agreement on the first survey were presented as a table with no rating required. Items that met criteria for non-consensus and newly proposed items were rerated.

We planned to administer a third survey, personalized for each panel member, to present their ratings and the group mean ratings on the second survey.

For each survey, the planning committee used the distribution of ratings to group items into inclusion, exclusion, and non-consensus categories according to the pre-defined criteria. Item categories and mean values were shared via email with the project team including the panel.

### Initial model development

Five team members with experience in economic evaluation and decision modeling who were not on the Delphi panel reviewed the list of health states and events for inclusion from the online modified Delphi process. This group met in June 2020 to discuss and draft a model structure.

### Face validity

The project team met virtually to assess the face validity of the proposed model structure and assumptions. Face validity, an important and iterative step in the model development process, is assessed by clinical experts to determine whether the structure, pathways, and assumptions of a model accurately reflect clinical and scientific understanding of the condition [30, 31]. Prior to the virtual meeting, a walk-through of the model including audio was created and circulated using Prezi, a presentation software (https://prezi.com/l__rx5dnv6i3/final_model-intro-and-pathway/?utm_campaign=share&utm_medium=copy) [32]. The model walk-through reiterated relevant concepts of economic evaluations first introduced in the surveys and described the target population, states, and transitions. Following circulation of the proposed model structure and Prezi walk-through, meetings were held in July 2020 with the project team to present survey results and discuss the proposed model structure. Feedback on whether the model appropriately represents the clinical pathway of community-dwelling older adults at risk of falling was obtained to assess the face validity of the model structure. Targeted clinical questions were asked during the meetings to assess face validity of the model. Questions were asked about the model pathway, fall-related injuries, and model assumptions.

### Ethics

This study received an exemption from the St. Michael’s Hospital Research Ethics Board because all participants and panel members were members of the research team.

## Results

### Literature review

#### Economic evaluations

During the economic evaluation search the information specialist identified a systematic review on economic evaluations of fall prevention interventions published in 2018 [33]. Consequently, we limited our screening to publication dates after 2017 to identify studies that could not have been included by the previous systematic review. We identified 13 economic evaluations that used state-transition models, including 9 that were included in the identified systematic review [4–6, 9, 10, 12–15] and 4 additional economic evaluations [7, 8, 11, 16] (S1 Table).

Most models (8/13, 62%) defined states based on a combination of fall risk and setting [4–8, 10, 15, 16], one model separated states by fall with or without fear of falling [12], three economic evaluations defined states based on fractures [11, 13, 14] and one defined states based solely on fall risk [6], although this model was for a population already living in residential aged care.

#### Clinical Practice Guidelines

The initial search resulted in 399 articles. Thirty-three articles were included for full-text screening. During the screening process, guidelines by the Registered Nurses’ Association of Ontario were found [34]. These guidelines were comprehensive and Ontario-focused and became the main source of information for the initial set of risk factors. In the guidelines, risk factors for falls were presented in four categories: behavioral or psychological; biological; environmental or situational, socio-economic. The guidelines also included a list of health conditions associated with an increased risk of falls or fall injuries, which formed the initial set of patient attributes in the first survey.

### Delphi process

#### Panel members

Eleven of the 15 individuals who were engaged in the project and not on the planning committee agreed to participate in the modified Delphi process. Panelists included seven members from Ontario, three from Alberta, and one from British Columbia. The panel comprised four clinicians, four geriatricians, one patient partner, six policy-makers, one health service researcher, one clinical operational leader, and two health economists with most panelists representing more than one area of expertise.

#### Survey results

The response rates were 100% (11/11) and 91% (10/11) on the first and second surveys, respectively. We deviated from our protocol and did not administer a third survey due to time constraints related to the COVID-19 pandemic.

*First survey*. All eleven panelists responded to the first survey and the distribution of ratings is shown in Tables 2 and 3. Using the pre-determined criteria for high agreement, the following 9 out of 20 possible health states/events met criteria for inclusion: hip fracture; surgery for hip fracture; head injury; fall; long-term care housing; vertebral fracture; hospitalization; rehabilitation hospitalization; and specialized dementia care or memory care in supportive housing. The following 18 patient attributes out of 38 met inclusion criteria: gait, balance, or mobility difficulties; history of falls/ previous falls; impaired vision; age, older age; dementia/cognitive impairment; physical inactivity; fear of falling; substance use; use of certain medications (anticonvulsants, tranquilizers, antihypertensives, opioids/narcotics); need for transfer assistance; home hazards (e.g., loose carpets, pets, stairs); use of restraints; overall frailty, older age; Parkinson’s disease; stroke; dementia/cognitive impairment; multiple sclerosis; and osteoporosis.

**Table 2:** Summary of health states/events ratings from the first Delphi survey.

	Panel Average Rating	Distribution of ratings (%)						
		No impact (0)	Very weak impact (1)	Weak impact (2)	Moderate impact (3)	Strong impact (4)	Very strong impact (5)	Don’t know
**Health states/events that met inclusion criteria**								
**Hip fracture**	4.89	0 (0%)	0 (0%)	0 (0%)	0 (0%)	1 (9%)	8 (73%)	2 (18%)
**Surgery for hip fracture**	4.80	0 (0%)	0 (0%)	0 (0%)	0 (0%)	2 (18%)	8 (73%)	1 (9%)
**Head injury**	4.70	0 (0%)	0 (0%)	0 (0%)	1 (9%)	1 (9%)	8 (73%)	1 (9%)
**Fall**	4.60	0 (0%)	0 (0%)	0 (0%)	1 (9%)	2 (18%)	7 (64%)	1 (9%)
**Long-term care housing (e.g., nursing home)**	4.40	0 (0%)	0 (0%)	1 (9%)	0 (0%)	3 (27%)	6 (55%)	1 (9%)
**Vertebral fracture**	4.40	0 (0%)	0 (0%)	1 (9%)	1 (9%)	1 (9%)	7 (64%)	1 (9%)
**Hospitalization**	4.40	0 (0%)	0 (0%)	1 (9%)	1 (9%)	1 (9%)	7 (64%)	1 (9%)
**Rehabilitation hospitalization**	4.40	0 (0%)	0 (0%)	0 (0%)	0 (0%)	6 (55%)	4 (36%)	1 (9%)
**Specialized dementia care or memory care in Supportive housing (e.g., retirement home)**	4.20	0 (0%)	0 (0%)	1 (9%)	1 (9%)	3 (27%)	5 (45%)	1 (9%)
**Health states/events that did not meet inclusion or exclusion criteria (non-consensus)**								
**Post-fall**	4.20	0 (0%)	0 (0%)	1 (9%)	2 (18%)	1 (9%)	6 (55%)	1 (9%)
**Wrist fracture**	4.00	0 (0%)	0 (0%)	0 (0%)	4 (36%)	2 (18%)	4 (36%)	1 (9%)
**Emergency department visit**	4.00	0 (0%)	1 (9%)	0 (0%)	2 (18%)	2 (18%)	5 (45%)	1 (9%)
**Independent housing (e.g., own home)**	3.70	1 (9%)	0 (0%)	0 (0%)	2 (18%)	4 (36%)	3 (27%)	1 (9%)
**Independent supported living service in Supportive housing**	3.70	0 (0%)	0 (0%)	0 (0%)	4 (36%)	5 (45%)	1 (9%)	1 (9%)
**Assisted living in Supportive housing (e.g., retirement home)**	3.70	0 (0%)	0 (0%)	0 (0%)	4 (36%)	5 (45%)	1 (9%)	1 (9%)
**Death due to fall**	3.73	1 (9%)	2 (18%)	0 (0%)	0 (0%)	1 (9%)	7 (64%)	0 (0%)
**Short term stay in Supportive housing (e.g., retirement home)**	3.50	0 (0%)	0 (0%)	0 (0%)	6 (55%)	3 (27%)	1 (9%)	1 (9%)
**Death**	3.55	1 (9%)	2 (18%)	0 (0%)	1 (9%)	1 (9%)	6 (55%)	0 (0%)
**Fear of falling**	3.50	0 (0%)	0 (0%)	2 (18%)	4 (36%)	1 (9%)	3 (27%)	1 (9%)
**No fall history**	2.90	1 (9%)	2 (18%)	1 (9%)	2 (18%)	1 (9%)	3 (27%)	1 (9%)

**Table 3:** Summary of patient attributes ratings from the first Delphi survey.

		Distribution of ratings (%)						
	Panel Average Rating	No association (0)	Very weak association (1)	Weak association (2)	Neutral association (3)	Strong association (4)	Very strong association (5)	Don’t know
**Patient attributes that met inclusion criteria**								
**Gait, balance, or mobility difficulties**	5.00	0 (0%)	0 (0%)	0 (0%)	0 (0%)	0 (0%)	10 (91%)	1 (9%)
**History of falls/previous falls**	4.80	0 (0%)	0 (0%)	0 (0%)	0 (0%)	2 (18%)	8 (73%)	1 (9%)
**Impaired vision**	4.50	0 (0%)	0 (0%)	0 (0%)	1 (9%)	3 (27%)	6 (55%)	1 (9%)
**Age, older age**	4.36	0 (0%)	0 (0%)	0 (0%)	1 (9%)	5 (45%)	5 (45%)	0 (0%)
**Dementia/cognitive impairment**	4.40	0 (0%)	0 (0%)	0 (0%)	1 (9%)	4 (36%)	5 (45%)	1 (9%)
**Physical inactivity**	4.60	0 (0%)	0 (0%)	0 (0%)	0 (0%)	4 (36%)	6 (55%)	1 (9%)
**Fear of falling**	4.22	0 (0%)	0 (0%)	0 (0%)	1 (9%)	5 (45%)	3 (27%)	2 (18%)
**Substance use**	4.10	0 (0%)	0 (0%)	0 (0%)	2 (18%)	5 (45%)	3 (27%)	1 (9%)
**Use of certain medications (anticonvulsants, tranquilizers, antihypertensives, opioids/narcotics)**	4.80	0 (0%)	0 (0%)	0 (0%)	0 (0%)	2 (18%)	8 (73%)	1 (9%)
**Need for transfer assistance**	4.40	0 (0%)	0 (0%)	0 (0%)	0 (0%)	6 (55%)	4 (36%)	1 (9%)
**Home hazards (e.g., loose carpets, pets, stairs)**	4.30	0 (0%)	0 (0%)	0 (0%)	1 (9%)	5 (45%)	4 (36%)	1 (9%)
**Use of restraints**	4.20	0 (0%)	0 (0%)	1 (9%)	0 (0%)	5 (45%)	4 (36%)	1 (9%)
**Overall frailty, older age**	4.60	0 (0%)	0 (0%)	0 (0%)	0 (0%)	4 (36%)	6 (55%)	1 (9%)
**Parkinson’s disease**	4.60	0 (0%)	0 (0%)	0 (0%)	0 (0%)	4 (36%)	6 (55%)	1 (9%)
**Stroke**	4.60	0 (0%)	0 (0%)	0 (0%)	0 (0%)	4 (36%)	6 (55%)	1 (9%)
**Dementia/cognitive impairment**	4.50	0 (0%)	0 (0%)	0 (0%)	0 (0%)	5 (45%)	5 (45%)	1 (9%)
**Multiple sclerosis**	4.44	0 (0%)	0 (0%)	0 (0%)	0 (0%)	5 (45%)	4 (36%)	2 (18%)
**Osteoporosis**	3.88	0 (0%)	1 (9%)	0 (0%)	0 (0%)	5 (45%)	2 (18%)	3 (27%)
**Patient attributes that did not meet inclusion or exclusion criteria (non-consensus)**								
**Malnutrition and related sarcopenia**	3.88	0 (0%)	0 (0%)	0 (0%)	3 (27%)	3 (27%)	2 (18%)	3 (27%)
**Sex**	3.43	0 (0%)	0 (0%)	0 (0%)	5 (45%)	1 (9%)	1 (18%)	4 (36%)
**Incontinence**	3.33	0 (0%)	0 (0%)	1 (9%)	4 (36%)	4 (36%)	0 (0%)	2 (18%)
**Hurrying, not paying attention**	3.80	0 (0%)	0 (0%)	0 (0%)	4 (36%)	4 (36%)	2 (18%)	1 (9%)
**Incorrect use of assistive devices**	3.80	0 (0%)	0 (0%)	0 (0%)	4 (36%)	4 (36%)	2 (18%)	1 (9%)
**Dual tasking**	3.67	0 (0%)	0 (0%)	1 (9%)	3 (27%)	3 (27%)	2 (18%)	2 (18%)
**Wearing unsupportive footwear**	3.67	0 (0%)	0 (0%)	0 (0%)	5 (45%)	2 (18%)	2 (18%)	2 (18%)
**Taking risks**	3.60	0 (0%)	0 (0%)	0 (0%)	4 (36%)	6 (55%)	0 (0%)	1 (9%)
**Gender**	2.67	0 (0%)	0 (0%)	3 (27%)	2 (18%)	1 (9%)	0 (0%)	5 (45%)
**Polypharmacy**	4.20	0 (0%)	0 (0%)	1 (9%)	2 (18%)	1 (9%)	6 (55%)	1 (9%)
**Prolonged hospital stay**	4.00	0 (0%)	0 (0%)	0 (0%)	2 (18%)	5 (45%)	2 (18%)	2 (18%)
**Side rails**	3.67	0 (0%)	0 (0%)	2 (18%)	2 (18%)	2 (18%)	3 (27%)	2 (18%)
**Unable to afford supportive footwear**	3.78	0 (0%)	0 (0%)	0 (0%)	3 (27%)	5 (45%)	1 (9%)	2 (18%)
**No social supports, isolated**	3.80	0 (0%)	0 (0%)	1 (9%)	2 (18%)	5 (45%)	2 (18%)	1 (9%)
**Unable to afford certain medications, nutritious food**	3.67	0 (0%)	0 (0%)	0 (0%)	4 (36%)	4 (36%)	1 (9%)	2 (18%)
**Unable to read**	3.00	1 (9%)	1 (9%)	1 (9%)	2 (18%)	2 (18%)	2 (18%)	2 (18%)
**Psychiatric illness (including depression)**	3.70	0 (0%)	0 (0%)	2 (18%)	2 (18%)	3 (27%)	3 (27%)	1 (9%)
**Osteoarthritis**	3.38	0 (0%)	0 (0%)	1 (9%)	3 (27%)	4 (36%)	0 (0%)	3 (27%)
**Cancer**	3.33	1 (9%)	0 (0%)	0 (0%)	3 (27%)	4 (36%)	1 (9%)	2 (18%)
**Hemophilia**	3.00	1 (9%)	0 (0%)	1 (9%)	1 (9%)	2 (18%)	1 (9%)	5 (45%)

Dementia/cognitive impairment was included as both a biological risk factor and a health condition and thus was rated by everyone twice. Although dementia/cognitive impairment met inclusion criteria both times it was rated, the rating differed slightly and it had a mean rating of 4.40 and 4.50 when rated as a biological factor and health condition, respectively (Table 3).

*Second survey*. The second survey asked about 11 health states/events that did not reach high agreement and 4 newly proposed health states/events. Twenty patient attributes that did not reach high agreement and three newly proposed patient attributes were also included. Almost all (10/11, 91%) panelists responded to the second survey and the distribution of ratings is shown in Tables 4 and 5. The following five health states/events met inclusion criteria in the second survey: post-fall; wrist fracture; emergency department visit; independent housing (e.g., own home); and death due to fall. The following eight patient attributes met inclusion criteria in the second survey: incorrect use of assistive devices; wearing unsupportive footwear; polypharmacy; prolonged hospital stay; unable to afford supportive footwear; no social supports, isolated; unable to afford certain medications, nutritious foods; and psychiatric illness (including depression).

**Table 4:** Summary of health states/events ratings from the second Delphi survey.

	Panel Average Rating	Distribution of ratings (%)						
		No impact (0)	Very weak impact (1)	Weak impact (2)	Moderate impact (3)	Strong impact (4)	Very strong impact (5)	Don’t know
**Health states/events that met inclusion criteria**								
**Post-fall**	4.38	0 (0%)	0 (0%)	0 (0%)	0 (0%)	5 (50%)	3 (30%)	2 (20%)
**Wrist fracture**	3.89	0 (0%)	0 (0%)	0 (0%)	1 (10%)	8 (80%)	0 (0%)	1 (10%)
**Emergency department visit**	4.10	0 (0%)	0 (0%)	0 (0%)	1 (10%)	7 (70%)	2 (20%)	0 (0%)
**Independent housing (e.g., own home)**	3.70	0 (0%)	0 (0%)	1 (10%)	1 (10%)	8 (80%)	0 (0%)	0 (0%)
**Death due to fall**	4.11	0 (0%)	0 (0%)	0 (0%)	1 (10%)	6 (60%)	2 (20%)	1 (10%)
**Health states/events that did not meet inclusion or exclusion criteria (non-consensus)**								
**Independent supported living service in Supportive housing**	3.70	0 (0%)	0 (0%)	0 (0%)	3 (30%)	7 (70%)	0 (0%)	0 (0%)
**Assisted living in Supportive housing (e.g., retirement home)**	3.70	0 (0%)	0 (0%)	0 (0%)	3 (30%)	7 (70%)	0 (0%)	0 (0%)
**Short term stay in Supportive housing (e.g., retirement home)**	3.40	0 (0%)	0 (0%)	0 (0%)	6 (60%)	4 (40%)	0 (0%)	0 (0%)
**Death**	3.78	0 (0%)	0 (0%)	0 (0%)	4 (40%)	3 (30%)	2 (20%)	1 (10%)
**Fear of falling**	3.44	0 (0%)	0 (0%)	0 (0%)	5 (50%)	4 (40%)	0 (0%)	1 (10%)
**No fall history**	2.60	0 (0%)	1 (10%)	3 (30%)	5 (50%)	1 (10%)	0 (0%)	0 (0%)
**Ankle fracture**	3.80	0 (0%)	0 (0%)	0 (0%)	4 (40%)	4 (40%)	2 (20%)	0 (0%)
**Humerus fracture**	3.89	0 (0%)	0 (0%)	0 (0%)	2 (20%)	6 (60%)	1 (10%)	1 (10%)
**Alternate level of care**	3.78	0 (0%)	0 (0%)	0 (0%)	4 (40%)	3 (30%)	2 (20%)	1 (10%)
**Transitional care unit**	3.78	0 (0%)	0 (0%)	0 (0%)	4 (40%)	3 (30%)	2 (20%)	1 (10%)

**Table 5:** Summary of patient attributes ratings from the second Delphi survey.

		Distribution of ratings (%)						
	Panel Average Rating	No association (0)	Very weak association (1)	Weak association (2)	Neutral association (3)	Strong association (4)	Very strong association (5)	Don’t know
**Patient attributes that met inclusion criteria**								
**Incorrect use of assistive devices**	4.00	0 (0%)	0 (0%)	0 (0%)	1 (10%)	8 (80%)	1 (10%)	0 (0%)
**Wearing unsupportive footwear**	4.00	0 (0%)	0 (0%)	0 (0%)	0 (0%)	9 (90%)	0 (0%)	1 (10%)
**Polypharmacy**	4.30	0 (0%)	0 (0%)	0 (0%)	0 (0%)	7 (70%)	3 (30%)	0 (0%)
**Prolonged hospital stay**	4.10	0 (0%)	0 (0%)	0 (0%)	0 (0%)	9 (90%)	1 (10%)	0 (0%)
**Unable to afford supportive footwear**	3.89	0 (0%)	0 (0%)	0 (0%)	1 (10%)	8 (80%)	0 (0%)	1 (10%)
**No social supports, isolated**	4.00	0 (0%)	0 (0%)	0 (0%)	0 (0%)	10 (100%)	0 (0%)	0 (0%)
**Unable to afford certain medications, nutritious food**	3.90	0 (0%)	0 (0%)	0 (0%)	1 (10%)	9 (90%)	0 (0%)	0 (0%)
**Psychiatric illness (including depression)**	3.80	0 (0%)	0 (0%)	0 (0%)	2 (20%)	8 (80%)	0 (0%)	0 (0%)
**Patient attributes that did not meet inclusion or exclusion criteria (non-consensus)**								
**Malnutrition and related sarcopenia**	3.89	0 (0%)	0 (0%)	0 (0%)	2 (20%)	6 (60%)	1 (10%)	1 (10%)
**Sex**	3.30	0 (0%)	0 (0%)	0 (0%)	7 (70%)	3 (30%)	0 (0%)	0 (0%)
**Incontinence**	3.40	0 (0%)	0 (0%)	0 (0%)	6 (60%)	4 (40%)	0 (0%)	0 (0%)
**Hurrying, not paying attention**	3.78	0 (0%)	0 (0%)	0 (0%)	2 (20%)	7 (70%)	0 (0%)	1 (10%)
**Dual tasking**	3.56	0 (0%)	0 (0%)	0 (0%)	4 (40%)	5 (50%)	0 (0%)	1 (10%)
**Taking risks**	3.78	0 (0%)	0 (0%)	0 (0%)	2 (20%)	7 (70%)	0 (0%)	1 (10%)
**Gender**	2.90	0 (0%)	0 (0%)	2 (20%)	7 (70%)	1 (10%)	0 (0%)	0 (0%)
**Side rails**	3.78	0 (0%)	0 (0%)	0 (0%)	2 (20%)	7 (70%)	0 (0%)	1 (10%)
**Unable to read**	3.30	0 (0%)	0 (0%)	1 (10%)	5 (50%)	4 (40%)	0 (0%)	0 (0%)
**Osteoarthritis**	3.40	0 (0%)	0 (0%)	0 (0%)	6 (60%)	4 (40%)	0 (0%)	0 (0%)
**Cancer**	3.20	0 (0%)	0 (0%)	0 (0%)	9 (90%)	0 (0%)	1 (10%)	0 (0%)
**Hemophilia**	3.10	0 (0%)	0 (0%)	0 (0%)	9 (90%)	1 (10%)	0 (0%)	0 (0%)
**Diabetes**	3.70	0 (0%)	0 (0%)	0 (0%)	4 (40%)	5 (50%)	1 (10%)	0 (0%)
**Cardiac disease**	3.40	0 (0%)	0 (0%)	1 (10%)	5 (50%)	3 (30%)	1 (10%)	0 (0%)
**Hypertension**	3.40	0 (0%)	0 (0%)	0 (0%)	6 (60%)	4 (40%)	0 (0%)	0 (0%)

### Initial model development

There were 14 health states/events selected for inclusion after both surveys (Table 6). Using these results, a proposed model structure was developed with members of the planning committee and discussions with a geriatrician with decision modeling experience and a health economist.

**Table 6:** Included health states/events as selected by Delphi panel.

Health state/event
Independent housing (e.g., own home)
Specialized dementia care or memory care in Supportive housing (e.g., retirement home)
Long-term care housing (e.g., nursing home)
Fall
Post-fall
Hip fracture
Wrist fracture
Vertebral fracture
Head injury
Emergency department visit
Hospital
Surgery for hip fracture
Rehabilitation hospital
Death due to fall

**Fig 1:**
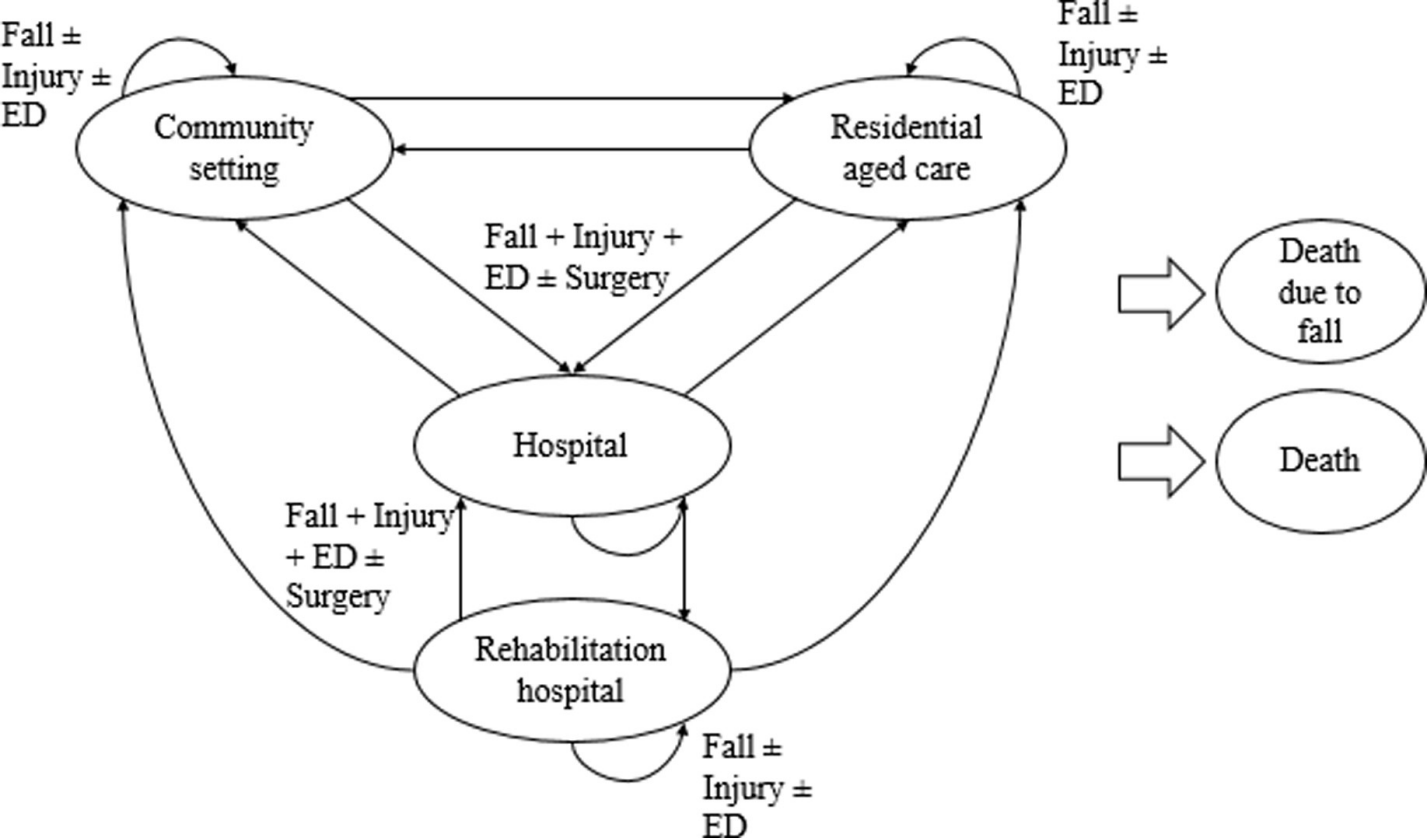
Model structure. Community setting: includes people who are living in settings such as their own home, retirement home, supportive housing or assisted living; Residential aged care setting: includes people living in settings such as long-term care facilities, nursing homes or residential care; Hospital: includes hospital inpatient Rehabilitation hospital: includes rehabilitation hospital inpatients; Death due to a fall: represents those who died as a result of a fall; Death: represents those who died by any cause other than a fall; ED: emergency department visit; Injury: includes hip fracture, vertebral fracture, wrist fracture, and head injury; Surgery: hip fracture surgery.

It was not possible to use the resulting list of health states/events as model health states since they would not meet the criteria of mutual exclusivity. For example, someone could have a hip fracture and be hospitalized. We chose to use a subset of health states that represent settings for our model’s states, which ensured mutual exclusivity and aligned with many previous studies that used community setting (independent housing) and residential aged care (long-term care housing) states [4, 5, 7–11, 15, 16]. All of the fall prevention interventions of interest occur in the community setting so another advantage of using settings as health states is that this setup simplifies the modelling of the interventions; everyone at the start of the model will be in the community setting and receiving one of seven interventions. Hospital and rehabilitation hospital were included as health states since they represent settings in which people may stay for longer periods of time. Specialized dementia care or memory care in Supportive housing (e.g., retirement home) was not included as a health state because data on such a specialized setting is limited. Additionally, other specialized settings were not included and it would have been inconsistent to add complexity for only one setting. Instead the independent housing setting was broadened to a community setting state including own home, retirement homes, supportive housing and assisted living. Furthermore, dementia/cognitive impairment met inclusion criteria in the patient attribute question. Although not selected for inclusion by the Delphi panel, death was included as a health state to ensure states were exhaustive (Fig 1). Many of the previous models also separated health states into low-, medium-, and high-risk fallers based on previous falls and fall injuries [4–9, 16]. Only two of the previous models included medical contact or treatment as part of the risk criteria [8, 16]. Because we used a microsimulation model, we had the ability to track previous falls, fall injuries, medical contact (such as emergency department visits), and treatments (such as surgery). This allowed us to use a reduced number of health states without sacrificing detail [27]. Based on the results from the surveys, the following events will be tracked: fall; hip fracture; vertebral fracture; wrist fracture; head injury; surgery for hip fracture; and emergency department visit. To account for other fall-related injuries, we added “minor injury” based on recommendations by Schwenk and colleagues on reporting fall injuries in randomized controlled trials [35], and fall-related injuries reported in the Canadian Community Health Survey, an annual, cross-sectional survey that collects information on health status, health care utilization, and health determinants for the Canadian population [36].

### Face validity

Summary results of the modified Delphi process were emailed to the project team including panel members after the first and second surveys. Subsequently, three meetings were held via Zoom [37] in July 2020 to relay findings, discuss the proposed model structure and assess its face validity. Experts who participated in face validity meetings included clinician-researchers with active practices, decades of experience, and over one hundred peer-reviewed publications. Between eight to ten people attended each meeting.

#### Pathway

Specific questions about the clinical pathways in the model were asked such as how an individual in Canada may navigate the health system after a fall. For example, admission to hospital for someone living in a community setting would be through the emergency department. Accordingly, our model pathway reflects this healthcare service use and cost.

#### Injuries

During the meetings, the potential fall-related injuries were expanded to include moderate injuries. Definitions of injuries were clarified such that a head injury refers specifically to an intracranial bleed, moderate injuries are dislocations or fractures not already included and minor injuries include soft tissue injuries such as cuts, scrapes, bruises, and sprains.

#### Model assumptions

The main assumption of our model imposed by the structure is that there can be a maximum of one fall per cycle and one injury per fall. Discussions with clinical experts revealed that for some frequent fallers, the assumption of one fall per two weeks is fallacious; however, the assumption is reasonable when considering the average number of falls for our target population. Our results may not apply to the small population of people who are very frequent fallers. The assumption about one injury per fall was highlighted as a limitation since people may sustain more than one injury in a fall event; however, Canadian data showed only 1.9% of older adults reported multiple injuries due to a fall [36]. Given the less frequent nature of multiple injuries, the decision was made to proceed with the given structure and highlight the assumption as a limitation.

## Discussion

A model conceptualization process was undertaken to develop a state-transition model for an economic evaluation of fall prevention interventions. Over twenty people contributed to model conceptualization process including eleven comprising the Delphi panel. Twenty-three health states/events and 41 patient attributes were rated. Of those, 14 health states/events and 26 patient attributes met high agreement criteria for inclusion.

The model structure used 5 of the 14 health states/events as health states (community setting; residential aged care setting; hospitalization; rehabilitation hospitalization; and death due to a fall) and 7 as events (fall, hip fracture, vertebral fracture, wrist fracture, head injury, surgery for hip fracture, and emergency department visit). During the Delphi process death was not selected by the panel although the proposed model time horizon was over the lifetime. Discussions later revealed potential reasons for the low ratings such as no events following death, interpreting death as an end point rather than an outcome in itself, and emphasis placed on morbidity in terms of quality of life and function rather than mortality in frail older adults who may have a short life expectancy.

The specific fracture outcomes (hip, vertebral, and wrist) that were identified as important by our Delphi panel were consistent with the recommended injurious outcomes in guidelines on conducting falls CEAs [18]. Another outcome listed by the guidelines was traumatic brain injury. Although our Delphi panel selected “head injury” for inclusion, this was later clarified to mean intracranial bleed by our geriatricians because an intracranial bleed is a more severe subtype of traumatic brain injuries.

Health economic modeling guidelines recommend developing a model without consideration of data availability, thus all patient attributes identified through the literature review were included on the surveys [20]. In total, 26 patient attributes met criteria for inclusion in our model. Once we obtained the list of patient attributes, data on some variables (such as wearing unsupportive footwear or incorrect use of assistive devices) were not available in the databases. Furthermore, many existing resources address one risk factor at a time which prevents us from incorporating all risk factors in the presence of each other. Therefore, the final list of patient attributes to be included in the model were those which were believed to be relevant and had available data.

This work was strengthened by a greater than 90% response rate on both surveys and the continual engagement of multiple end user partners throughout the process. There were limitations that should be considered when interpreting the results. First, we did not conduct a systematic review of the literature on economic evaluations of falls. We limited our searches to languages comprehended by the planning committee (English and Thai), and risk factors searches to clinical practice guidelines. This may have resulted in relevant health states, events, and patient attributes being excluded from input into the surveys. Second, our Delphi panel did not achieve consensus on all items and a third survey was not completed. We chose not to prepare and circulate a third survey due to time constraints on the planning committee and panel members related to the COVID-19 pandemic [38]. Additional items may have reached high agreement for inclusion with additional surveys and may have resulted in changes to our proposed model structure. The use of “Don’t Know” which was intended to dissuade uninformative responses, was used less frequently in the second compared to the first survey. One major difference between the first and second survey was the inclusion of the panel average from the first survey on the second. Many respondents who selected don’t know on the first survey provided a response on the second survey consistent with the panel average. Finally, our Delphi panel included 11 people living in mainly urban settings in 3 Canadian provinces who may not be reflective of the diversity of the Canadian population nor an international perspective. Furthermore, we included limited specialists on our panel and the inclusion of other specialists such as physiotherapists, occupational therapists, and kinesiologists, and other panels may produce different results.

There were limitations to our process that should be considered by others embarking on similar work. Unlike other studies that underwent modified Delphi processes, we drafted a state-transition model rather than a conceptual model [21, 22, 24]. Furthermore, aligned with Afzali and colleagues’ work [21], we engaged our Delphi panel after the literature search such that the panel rated all items independently of each other. This contrasted with other methods in which a conceptual model was first drafted and then the panel had the opportunity to comment on the importance of items but also how they relate to the overall picture [22, 24]. This method could have been a more informative approach to determine the clinical pathway of patients.

## Conclusions

This study demonstrated a transparent model conceptualization process, which resulted in a six-state microsimulation model. Model conceptualization is a crucial step in economic evaluations using decision modeling, but documentation and examples of the process is lacking. The dissemination of this work allows others who are undergoing similar work to build on our process. The transparency of the model development allows others to determine the reasonableness of the assumptions we used and determine for themselves whether this work or a similar approach would be appropriate for their decision problem.

## Supporting information

S1 File Economic evaluation search strategy. (PDF)

S2 File Clinical practice guidelines search strategy. (PDF)

S3 File Survey 1. (PDF)

S4 File Survey 2. (PDF)

S1 Table Summary of model-based cost-effectiveness analyses. (DOCX)
