# Supplementary material for: Developing a fall prevention intervention economic model
Source: PLoS One. 2023 Jan 27;18(1):e0280572. doi: 10.1371/journal.pone.0280572 (PMC9882648; doi:10.1371/journal.pone.0280572)
Supplement: S1 File — (PDF) [file pone.0280572.s001.pdf]

Database: Ovid MEDLINE: Epub Ahead of Print, In-Process & Other Non-Indexed Citations, Ovid MEDLINE® Daily and Ovid MEDLINE® <1946-Present>

Search Strategy:

----

1 Accidental Falls/pc [Prevention & Control] (8267)
2 ((fall or falls or falling) adj3 prevent*).tw,kf. (6564)
3 ((fall or falls or falling) adj3 reduc*).tw,kf. (3054)
4 ((fall or falls or falling) adj3 decreas*).tw,kf. (1133)
5 ((fall or falls or falling) adj3 protect*).tw,kf. (407)
6 ((slip or slips or slipping) adj3 prevent*).tw,kf. (250)
7 or/1-6 (14380)
8 Economics/ (27028)
9 exp "costs and cost analysis"/ (224438)
10 Economics, Dental/ (1902)
11 exp economics, hospital/ (23546)
12 Economics, Medical/ (9010)
13 Economics, Nursing/ (3986)
14 Economics, Pharmaceutical/ (2857)
15 (economic$ or cost or costs or costly or costing or price or prices or pricing or pharmacoeconomic$).ti,ab. (719539)
16 (expenditure$ not energy).ti,ab. (27357)
17 value for money.ti,ab. (1549)
18 budget$.ti,ab. (27186)
19 or/8-18 (866184)
20 ((energy or oxygen) adj cost).ti,ab. (3868)
21 (metabolic adj cost).ti,ab. (1312)
22 ((energy or oxygen) adj expenditure).ti,ab. (23451)
23 or/20-22 (27691)
24 19 not 23 (859813)
25 letter.pt. (1026559)
26 editorial.pt. (490082)
27 historical article.pt. (351477)
28 exp animals/ not humans/ (4578616)
29 25 or 26 or 27 or 28 (6373984)
30 24 not 29 [NHS EED MEDLINE Search Filter http://www.crd.york.ac.uk/crdweb/searchstrategies.asp#nhseedmedline] (773349)
31 7 and 30 (1267)
32 limit 7 to "economics (best balance of sensitivity and specificity)" (1149)
33 limit 7 to "costs (best balance of sensitivity and specificity)" (801)
34 31 or 32 or 33 (1303)
35 limit 34 to ("all child (0 to 18 years)" or "young adult (19 to 24 years)") (117)

36 limit 35 to "all aged (65 and over)" (59)
37 35 not 36 (58)
38 34 not 37 (1245)
39 limit 38 to (comment or editorial or historical article or letter) (15)
40 38 not 39 (1230)
41 limit 40 to (english or thai) (1164)
42 remove duplicates from 41 (1159)

Database: Embase Classic+Embase <1947 to 2019 May 10>
Search Strategy:
----
1 falling/pc [Prevention] (2928)
2 ((fall or falls or falling) adj3 prevent*).tw. (8694)
3 ((fall or falls or falling) adj3 reduc*).tw. (4263)
4 ((fall or falls or falling) adj3 decreas*).tw. (1813)
5 ((fall or falls or falling) adj3 protect*).tw. (507)
6 ((slip or slips or slipping) adj3 prevent*).tw. (362)
7 fall risk/ and (prevent* or reduc* or decreas*).tw. (2053)
8 1 or 2 or 3 or 4 or 5 or 6 or 7 (16227)
9 Health Economics/ (36670)
10 exp Economic Evaluation/ (288256)
11 exp Health Care Cost/ (276603)
12 pharmacoeconomics/ (7007)
13 9 or 10 or 11 or 12 (513022)
14 (econom$ or cost or costs or costly or costing or price or prices or pricing or pharmacoeconomic$).ti,ab. (983203)
15 (expenditure$ not energy).ti,ab. (37870)
16 (value adj2 money).ti,ab. (2272)
17 budget$.ti,ab. (36170)
18 14 or 15 or 16 or 17 (1017870)
19 13 or 18 (1238170)
20 letter.pt. (1063587)
21 editorial.pt. (600043)
22 note.pt. (751094)
23 20 or 21 or 22 (2414724)
24 19 not 23 (1141175)
25 (metabolic adj cost).ti,ab. (1454)
26 ((energy or oxygen) adj cost).ti,ab. (4341)
27 ((energy or oxygen) adj expenditure).ti,ab. (30311)
28 25 or 26 or 27 (35015)
29 24 not 28 (1133862)

30 animal/ (1904951)
31 exp animal experiment/ (2380598)
32 nonhuman/ (5786898)
33 (rat or rats or mouse or mice or hamster or hamsters or animal or animals or dog or dogs or cat or cats or bovine or sheep).ti,ab,sh. (6310479)
34 30 or 31 or 32 or 33 (9276766)
35 exp human/ (20954171)
36 human experiment/ (451315)
37 35 or 36 (20955766)
38 34 not (34 and 37) (6952058)
39 29 not 38 [NHS EED Ovid EMBASE Economics Search Filter http://www.crd.york.ac.uk/crdweb/searchstrategies.asp] (1032560)
40 8 and 39 (1581)
41 limit 40 to child <unspecified age> (51)
42 limit 41 to aged <65+ years> (25)
43 41 not 42 (26)
44 40 not 43 (1555)
45 limit 44 to embase (683)
46 limit 45 to (english or thai) (639)
47 remove duplicates from 46 (627)

Database: EBM Reviews - NHS Economic Evaluation Database <1st Quarter 2016>
Search Strategy:

----
1 Accidental Falls/pc [Prevention & Control] (34)
2 ((fall or falls or falling) adj3 prevent*).tw. (35)
3 ((fall or falls or falling) adj3 reduc*).tw. (17)
4 ((fall or falls or falling) adj3 decreas*).tw. (1)
5 ((fall or falls or falling) adj3 protect*).tw. (1)
6 ((slip or slips or slipping) adj3 prevent*).tw. (0)
7 1 or 2 or 3 or 4 or 5 or 6 (45)
8 limit 7 to "all child (0 to 18 years)" (1)
9 limit 8 to "all adult (19 plus years)" (0)
10 8 not 9 (1)
11 7 not 10 (44)

Database: EBM Reviews - Cochrane Central Register of Controlled Trials <April 2019>, EBM Reviews - Cochrane Database of Systematic Reviews <2005 to May 2, 2019>, EBM Reviews - Database of Abstracts of Reviews of Effects <1st Quarter 2016>, EBM Reviews - Cochrane Methodology Register <3rd Quarter 2012>, EBM Reviews - Health Technology Assessment <4th Quarter 2016>
Search Strategy:

----

1 Accidental Falls/pc [Prevention & Control] (4)
2 ((fall or falls or falling) adj3 prevent*).tw. (1805)
3 ((fall or falls or falling) adj3 reduc*).tw. (1765)
4 ((fall or falls or falling) adj3 decreas*).tw. (502)
5 ((fall or falls or falling) adj3 protect*).tw. (95)
6 ((slip or slips or slipping) adj3 prevent*).tw. (27)
7 1 or 2 or 3 or 4 or 5 or 6 (3338)
8 Economics/ (54)
9 exp "costs and cost analysis"/ (11012)
10 Economics, Dental/ or exp economics, hospital/ or Economics, Medical/ or Economics, Nursing/ or Economics, Pharmaceutical/ (792)
11 (economic$ or cost or costs or costly or costing or price or prices or pricing or pharmacoeconomic$).ti,ab. (69235)
12 (expenditure$ not energy).ti,ab. (1640)
13 value for money.ti,ab. (226)
14 budget$.ti,ab. (989)
15 8 or 9 or 10 or 11 or 12 or 13 or 14 (72039)
16 7 and 15 (412)
17 remove duplicates from 16 (397)

AgeLine

#	Query	Limiters/Expanders	Results
S15	S13 NOT S14	Search modes - Boolean/Phrase	225
S14	S13	Limiters - Publication Type: Book, Chapter, Dissertation, Video<br>Search modes - Boolean/Phrase	21
S13	S4 OR S12	Search modes - Boolean/Phrase	246
S12	S10 AND S11	Search modes - Boolean/Phrase	216
S11	economic* or cost or costs or costly or costing or price or prices or pricing or budget*	Search modes - Boolean/Phrase	29,716

S10	S5 OR S6 OR S7 OR S8 OR S9	Search modes - Boolean/Phrase	1,530
S9	((slip or slips or slipping) N3 prevent*)	Search modes - Boolean/Phrase	9
S8	((fall or falls or falling) N3 protect*)	Search modes - Boolean/Phrase	61
S7	((fall or falls or falling) N3 decreas*)	Search modes - Boolean/Phrase	160
S6	((fall or falls or falling) N3 reduc*)	Search modes - Boolean/Phrase	593
S5	(fall or falls or falling) N3 prevent*	Search modes - Boolean/Phrase	1,090
S4	S1 AND S2 AND S3	Search modes - Boolean/Phrase	36
S3	DE "Prevention" OR DE "Accident Prevention"	Search modes - Boolean/Phrase	1,702
S2	((DE "Cost Effectiveness" OR DE "Costs") AND (DE "Institutional Costs" OR DE "Hospital Costs" OR DE "Health Service Costs" OR DE "Drug Costs")) OR (DE "Quality of Life")	Search modes - Boolean/Phrase	6,514
S1	DE "Falls"	Search modes - Boolean/Phrase	2,642
