# Supplementary material for: Developing a fall prevention intervention economic model
Source: PLoS One. 2023 Jan 27;18(1):e0280572. doi: 10.1371/journal.pone.0280572 (PMC9882648; doi:10.1371/journal.pone.0280572)
Supplement: S2 File — (PDF) [file pone.0280572.s002.pdf]

Literature search on October 23, 2019 in Medline

1	((fall or falls or falling) adj3 prevent*).tw,kf	6796
2	((fall or falls or falling) adj3 reduc*).tw,kf.	3159
3	((fall or falls or falling) adj3 decreas*).tw,kf.	1162
4	((fall or falls or falling) adj3 protect*).tw,kf.	414
5	((slip or slips or slipping) adj3 prevent*).tw,kf.	254
6	Accidental Falls/pc [Prevention & Control]	8541
7	1 or 2 or 3 or 4 or 5 or 6	14837
8	exp clinical pathway/	6435
9	exp clinical protocol/	162275
10	exp consensus/	11453
11	exp consensus development conference/	11637
12	exp consensus development conferences as topic/	2761
13	critical pathways/	6435
14	exp guideline/	32796
15	guidelines as topic/	38654
16	exp practice guideline/	25940
17	practice guidelines as topic/	112924
18	health planning guidelines/	4060
19	(guideline or practice guideline or consensus development conference or consensus development conference, NIH).pt.	41914
20	(position statement* or policy statement* or practice parameter* or best practice*).ti,ab,kf,kw.	30586
21	(standards or guideline or guidelines).ti,kf,kw.	104535
22	((practice or treatment* or clinical) adj guideline*).ab.	37432
23	(CPG or CPGs).ti.	5544

24	consensus*.ti,kf,kw.	24400
25	consensus*.ab. /freq=2	23647
26	((critical or clinical or practice) adj2 (path or paths or pathway or pathways or protocol*)).ti,ab,kf,kw.	19035
27	recommendat*.ti,kf,kw.	38684
28	(care adj2 (standard or path or paths or pathway or pathways or map or maps or plan or plans)).ti,ab,kf,kw.	54408
29	(algorithm* adj2 (screening or examination or test or tested or testing or assessment* or diagnosis or diagnoses or diagnosed or diagnosing)).ti,ab,kf,kw.	7095
30	(algorithm* adj2 (pharmacotherap* or chemotherap* or chemotreatment* or therap* or treatment* or intervention*)).ti,ab,kf,kw.	9201
31	4 or 8 or 9 or 10 or 11 or 12 or 13 or 14 or 15 or 16 or 17 or 18 or 19 or 20 or 21 or 22 or 23 or 24 or 25 or 26 or 27 or 28 or 29 or 30	586362
32	7 and 31	1238
33	limit 32 to "all aged (65 and over)"	596
34	limit 33 to english language	543
35	limit 34 to (female or humans or male)	543
36	(Randomized Controlled Trial or Controlled Clinical Trial or Pragmatic Clinical Trial or Equivalence Trial or Clinical Trial, Phase III).pt.	584187
37	Randomized Controlled Trial/	492634
38	exp Randomized Controlled Trials as Topic/	130993
39	"Randomized Controlled Trial (topic)"/	0
40	Controlled Clinical Trial/	93388
41	exp Controlled Clinical Trials as Topic/	136126
42	"Controlled Clinical Trial (topic)"/	0
43	Randomization/	100879
44	Random Allocation/	100879
45	Double-Blind Method/	154184
46	Double Blind Procedure/	0

47	Double-Blind Studies/	154184
48	Single-Blind Method/	27496
49	Single Blind Procedure/	0
50	Single-Blind Studies/	27496
51	Placebos/	34564
52	Placebo/	0
53	Control Groups/	1635
54	Control Group/	1635
55	(random* or sham or placebo*).ti,ab,hw,kf,kw.	1433449
56	((singl* or doubl*) adj (blind* or dumm* or mask*)).ti,ab,hw,kf,kw.	229241
57	((tripl* or trebl*) adj (blind* or dumm* or mask*)).ti,ab,hw,kf,kw.	915
58	(control* adj3 (study or studies or trial* or group*)).ti,ab,kf,kw.	935141
59	(Nonrandom* or non random* or non-random* or quasi-random* or quasirandom*).ti,ab,hw,kf,kw.	41981
60	allocated.ti,ab,hw.	62140
61	((open label or open-label) adj5 (study or studies or trial*)).ti,ab,hw,kf,kw.	32676
62	((equivalence or superiority or non-inferiority or noninferiority) adj3 (study or studies or trial*)).ti,ab,hw,kf,kw.	7497
63	(pragmatic study or pragmatic studies).ti,ab,hw,kf,kw.	369
64	((pragmatic or practical) adj3 trial*).ti,ab,hw,kf,kw.	4466
65	((quasiexperimental or quasi-experimental) adj3 (study or studies or trial*)).ti,ab,hw,kf,kw.	7003
66	(phase adj3 (III or "3") adj3 (study or studies or trial*)).ti,hw,kf,kw.	26539
67	36 or 37 or 38 or 39 or 40 or 41 or 42 or 43 or 44 or 45 or 46 or 47 or 48 or 49 or 50 or 51 or 52 or 53 or 54 or 55 or 56 or 57 or 58 or 59 or 60 or 61 or 62 or 63 or 64 or 65 or 66	2063477
68	35 not 67	399
