# Supplementary material for: Developing a fall prevention intervention economic model
Source: PLoS One. 2023 Jan 27;18(1):e0280572. doi: 10.1371/journal.pone.0280572 (PMC9882648; doi:10.1371/journal.pone.0280572)
Supplement: S3 File — (PDF) [file pone.0280572.s003.pdf]

Economic Impact of Falls Prevention Interventions - Model Conceptualization Survey #1

INTRODUCTION

Thank you for agreeing to participate in our modified Delphi process for the economic impact of falls prevention interventions project.

This process aims to assist in the construction a model that will simulate patients over their lifetime to estimate the cost-effectiveness of our identified falls prevention interventions through a series of online surveys.

For more information on the overall project please visit the link below containing background documents such as meeting summary #1 and the originally proposed model.
Background documents: [redacted].

OVERALL OBJECTIVE OF THE STUDY (AND MODEL)
We are aiming to create a model to represent the clinical pathway of elderly Canadians (≥ 65 years) at risk of falls and living in a community or residential care setting.

Consequently, we have three specific goals to aid with this process.

- **Goal 1:** to obtain high agreement (≥ 80%) on the set of health states and events to be included in our model.
- **Goal 2:** to obtain high agreement (≥ 80%) on the set of patient attributes associated with falls, costs, and quality of life to be included in our model.
- **Goal 3:** to establish face validity (i.e., whether the structure and pathways of the model accurately reflect the clinical pathways of our population and interventions) of the model structure [1].

As goals 1 and 2 of this process are to obtain high agreement, we may require multiple rounds (maximum being three) to reach these goals. Goal 3 will be addressed in later surveys.

We have created a planning committee comprised of Dr. Andrea Tricco, Dr. Ahmed Bayoumi, Lisa Masucci, Hailey Saunders, and Dr. Wanrudee Isaranuwatchai, to help develop the surveys, analyze, and disseminate the results.

Note that your opinions will be **anonymous** to others on the panel. The planning committee will send your individual responses back to you along with summary measures of the panel's responses. Therefore, your responses will not be anonymous to the planning committee; however, they will only be shared individually back to you and otherwise circulated as aggregated results.

We anticipate that all sections of this survey will take 15 - 20 minutes for you to complete.

1. Eddy, D.M., et al., Model transparency and validation: a report of the ISPOR-SMDM Modeling Good Research Practices Task Force–7. Medical Decision Making, 2012. 32(5): p. 733-743.

BACKGROUND INFORMATION ON MODELING IN HEALTH ECONOMICS

The following subsections introduce general concepts in health economics models which will be referred to in the questions we would like you to answer.

Specifically, the following concepts are discussed: 1) health states; 2) time horizon and cycle length; 3) events; and 4) cohort vs. individual-level models.

1) Model structure – health states
A clinical pathway or disease progression can be represented by different health states representing the possible conditions in which a person may be [2].

Health states must be: 1) mutually exclusive; and 2) exhaustive.

In the simplest model, we could have 3 health states: healthy, sick, or dead. We could depict that model as shown in Figure 1 below. The arrows in Figure 1 indicate the possible transitions (movement) between health states. For example, people in a “Healthy” state could move to a “Sick” state, and move back once recovered.

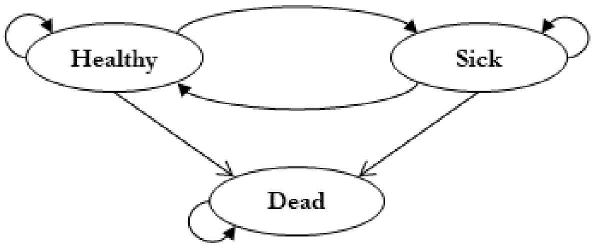

Figure 1 Simple State Transition Model

We want our model to be as simple as possible but still be able to capture important aspects related to our decision problem. Therefore, health states will be included if they have an impact on the costs, health-related quality of life, mortality, and progression to other states. In our example above, someone who is sick would have different costs and health-related quality of life compared to a healthy individual. Someone who is in the sick state would also be more likely to progress to the death state. We will combine both the health-related quality of life and mortality into quality-adjusted life years (QALYs).

2) Model structure – time horizon and cycle length

Other important features of the model are the **time horizon**, i.e., for how long we follow individuals, and **cycle length**, i.e., the amount of time spent in a health state before a possible transition.

Our model will have:

- Time horizon = lifetime
- Cycle length = one-month

3) Model structure – events

An **event** is something that may cause someone to move from one health state to another. It can have a cost and QALYs associated with it but it is short (i.e., lasts less than one cycle length, in our case it is something that lasts less than one-month).

Consider a fall as an example. A fall has associated costs and affects quality of life, but someone does not stay in a fall for a month. Therefore, we may choose to make a fall an event. On the other hand, there may be ongoing impacts on costs and reductions in quality of life after a fall and we may choose to make a post-fall health state instead.

4) Model structure – cohort vs. individual-level

Another important feature of the model is whether it is a **cohort or individual-level model**. A state-transition model can be used to simulate a cohort moving through the model or it can simulate individuals one at a time [2]. Individual-level models can keep track of each individual's history (e.g., fall history, medication use).

Our model will be an individual-level model (a.k.a. microsimulation model).

We have the ability to keep track of patient history in microsimulation models. For example, a person who has experienced a fall may be more likely to experience another fall. If this is the case, then in addition to including a fall as an event or health state we would also keep track of whether someone has fallen. If the time since fall or number of falls are important (i.e., modify cost, QALYs, and future transitions), then we could keep track of those too.

2. Siebert, U., et al., State-transition modeling: a report of the ISPOR-SMDM modeling good research practices task force–3. Medical Decision Making, 2012. 32(5): p. 690-700.

Economic Impact of Falls Prevention Interventions - Model Conceptualization Survey #1

SURVEY

SECTION 1

This section will help us achieve **Goal 1** (to obtain high agreement (≥ 80%) on the set of health states and events to be included in our model).

- **In question 1**, we will present you with a list of potential health states and events and ask you to score each option.
- **In question 2**, we will give you the opportunity to suggest addition health states and events not listed in question 1.

Note: Please score based on your experience, knowledge, and opinion. You are not expected to read or research to answer these questions; there is the option to answer "Don't Know".

* 1. **Potential health states and events**

Health states should be included if they have distinct costs, QALYs from other health states.

Events are shorter than one cycle (in our case, last less than one month), may have associated costs and QALYs and may cause someone to move from one health state to another. When selecting whether something should be a health state or event, consider whether there could be ongoing impacts on costs and QALYs.

Please score the following health states and events based on the strength of their impact (from very weak to very strong) on costs, QALYs, and progression to other health states specifically for older adults at risk of falls.

After each row there is a box in which you have the option to indicate whether you think the item should be a health state or event based on the definitions previously provided.

	**No** impact	**Very Weak** impact	**Weak** impact	**Moderate** impact	**Strong** impact	**Very Strong** impact	**Don't Know**
Independent housing (e.g., own home)	○	○	○	○	○	○	○
Health state or event? [ ]							
Independent supported living service in Supportive housing	○	○	○	○	○	○	○

	**No** impact	**Very Weak** impact	**Weak** impact	**Moderate** impact	**Strong** impact	**Very Strong** impact	**Don't Know**
Health state or event?							
Assisted living in Supportive housing (e.g., retirement home)							
Health state or event?							
Specialized dementia care or memory care in Supportive housing (e.g., retirement home)							
Health state or event?							
Short term stay in Supportive housing (e.g., retirement home)							
Health state or event?							
Long-term care housing (e.g., nursing home)							
Health state or event?							
No fall history							
Health state or event?							
Fall							
Health state or event?							
Post-fall							
Health state or event?							
Hip fracture							
Health state or event?							

	**No** impact	**Very Weak** impact	**Weak** impact	**Moderate** impact	**Strong** impact	**Very Strong** impact	**Don't Know**
Wrist fracture	○	○	○	○	○	○	○
Health state or event?							
Vertebral fracture	○	○	○	○	○	○	○
Health state or event?							
Head injury	○	○	○	○	○	○	○
Health state or event?							
Emergency department visit	○	○	○	○	○	○	○
Health state or event?							
Hospitalization	○	○	○	○	○	○	○
Health state or event?							
Surgery for hip fracture	○	○	○	○	○	○	○
Health state or event?							
Rehabilitation hospitalization	○	○	○	○	○	○	○
Health state or event?							
Fear of falling	○	○	○	○	○	○	○
Health state or event?							
Death due to fall	○	○	○	○	○	○	○
Health state or event?							
Death	○	○	○	○	○	○	○

	**No** impact	**Very Weak** impact	**Weak** impact	**Moderate** impact	**Strong** impact	**Very Strong** impact	**Don't Know**

Health state or event?

* 2. Are there any important (strong or very strong) health states or events that are missing?

- No
- Yes, please list

Economic Impact of Falls Prevention Interventions - Model Conceptualization Survey #1

SECTION 2

This section will help us achieve **Goal 2** (to obtain high agreement (≥ 80%) on the set of patient attributes associated with falls, costs, and quality of life to be included in our model).

- **In questions 3 - 7**, we will present you with a list of potential patient attributes and ask you to score each option.
- **In question 8**, we will ask you to list any important patient attributes that you feel were missing from questions 3 - 7.

Note: Please score based on your experience, knowledge, and opinion. You are not expected to read or research to answer these questions; there is the option to answer "Don't Know".

For definitions of sex and gender from CIHR, please visit: https://cihr-irsc.gc.ca/e/47830.html

* 3. **Potential patient attributes - biological factors**

Please score each patient attribute on the strength of its association with falls (from very weak [score 1] to very strong [score 5]) including the risk of a fall, risk of injury after a fall, type of injury after a fall, cost of treating an injury after a fall, disease progression, clinical pathway after a fall (e.g., treatment, hospitalization, rehabilitation hospitalization, admittance to long-term care), quality of life, resource use, and mortality. The list of patient attributes are categorized as they are in clinical best practice guidelines, Preventing Falls and Reducing Injury from Falls Fourth Edition [3].

This question focuses on biological factors.

	**No** association	**Very Weak** association	**Weak** association	**Moderate** association	**Strong** association	**Very Strong** association	**Don't Know**
Age, older age	○	○	○	○	○	○	○
History of falls/ Previous falls	○	○	○	○	○	○	○
Dementia/cognitive impairment	○	○	○	○	○	○	○
Gait, balance, or mobility difficulties	○	○	○	○	○	○	○
Impaired vision	○	○	○	○	○	○	○
Incontinence	○	○	○	○	○	○	○
Malnutrition and related sarcopenia	○	○	○	○	○	○	○
Sex	○	○	○	○	○	○	○

* 4. **Potential patient attributes - behavioural or psychological factors**

Please score each patient attribute on the strength of its association with falls (from very weak [score 1] to very strong [score 5]) including the risk of a fall, risk of injury after a fall, type of injury after a fall, cost of treating an injury after a fall, disease progression, clinical pathway after a fall (e.g., treatment, hospitalization, rehabilitation hospitalization, admittance to long-term care), quality of life, resource use, and mortality. The list of patient attributes are categorized as they are in clinical best practice guidelines, Preventing Falls and Reducing Injury from Falls Fourth Edition [3].

This question focuses on behavioural or psychological patient attributes.

	**No** association	**Very Weak** association	**Weak** association	**Moderate** association	**Strong** association	**Very Strong** association	**Don't Know**
Hurrying, not paying attention	◯	◯	◯	◯	◯	◯	◯
Taking risks	◯	◯	◯	◯	◯	◯	◯
Physical inactivity	◯	◯	◯	◯	◯	◯	◯
Fear of falling	◯	◯	◯	◯	◯	◯	◯
Dual tasking	◯	◯	◯	◯	◯	◯	◯
Incorrect use of assistive devices	◯	◯	◯	◯	◯	◯	◯
Wearing unsupportive footwear	◯	◯	◯	◯	◯	◯	◯
Substance use	◯	◯	◯	◯	◯	◯	◯
Gender	◯	◯	◯	◯	◯	◯	◯

* 5. **Potential patient attributes - environmental or situational factors**

Please score each patient attribute on the strength of its association with falls (from very weak [score 1] to very strong [score 5]) including the risk of a fall, risk of injury after a fall, type of injury after a fall, cost of treating an injury after a fall, disease progression, clinical pathway after a fall (e.g., treatment, hospitalization, rehabilitation hospitalization, admittance to long-term care), quality of life, resource use, and mortality. The list of patient attributes are categorized as they are in clinical best practice guidelines, Preventing Falls and Reducing Injury from Falls Fourth Edition [3].

	**No** association	**Very Weak** association	**Weak** association	**Moderate** association	**Strong** association	**Very Strong** association	**Don't Know**
Polypharmacy	○	○	○	○	○	○	○
Use of certain medications (anticonvulsants, tranquilizers, antihypertensives, opioids/narcotics)	○	○	○	○	○	○	○
Home hazards (e.g., loose carpets, pets, stairs)	○	○	○	○	○	○	○
Prolonged hospital stay	○	○	○	○	○	○	○
Need for transfer assistance	○	○	○	○	○	○	○
Use of restraints	○	○	○	○	○	○	○
Side rails	○	○	○	○	○	○	○

* 6. **Potential patient attributes - socioeconomic factors**

Please score each patient attribute on the strength of its association with falls (from very weak [score 1] to very strong [score 5]) including the risk of a fall, risk of injury after a fall, type of injury after a fall, cost of treating an injury after a fall, disease progression, clinical pathway after a fall (e.g., treatment, hospitalization, rehabilitation hospitalization, admittance to long-term care), quality of life, resource use, and mortality. The list of patient attributes are categorized as they are in clinical best practice guidelines, Preventing Falls and Reducing Injury from Falls Fourth Edition [3].

	**No** association	**Very Weak** association	**Weak** association	**Moderate** association	**Strong** association	**Very Strong** association	**Don't Know**
Unable to afford supportive footwear	○	○	○	○	○	○	○
Unable to afford certain medications, nutritious foods	○	○	○	○	○	○	○
No social supports, isolated	○	○	○	○	○	○	○
Unable to read	○	○	○	○	○	○	○

* 7. **Potential patient attributes - health conditions**

Please score each patient attribute on the strength of its association with falls (from very weak [score 1] to very strong [score 5]) including the risk of a fall, risk of injury after a fall, type of injury after a fall, cost of treating an injury after a fall, disease progression, clinical pathway after a fall (e.g., treatment, hospitalization, rehabilitation hospitalization, admittance to long-term care), quality of life, resource use, and mortality. The list of patient attributes are categorized as they are in clinical best practice guidelines, Preventing Falls and Reducing Injury from Falls Fourth Edition [3].

	**No** association	**Very Weak** association	**Weak** association	**Neutral** association	**Strong** association	**Very Strong** association	**Don't Know**
Cancer	○	○	○	○	○	○	○
Dementia/cognitive impairment	○	○	○	○	○	○	○
Hemophilia	○	○	○	○	○	○	○
Multiple sclerosis	○	○	○	○	○	○	○
Osteoarthritis	○	○	○	○	○	○	○
Osteoporosis	○	○	○	○	○	○	○
Overall frailty, older age	○	○	○	○	○	○	○
Parkinson’s disease	○	○	○	○	○	○	○
Psychiatric illness (including depression)	○	○	○	○	○	○	○
Stroke	○	○	○	○	○	○	○

* 8. **Are there any important (strong or very strong) patient attributes that are missing?**

○ No

○ Yes, please list

* 9. **Conflict of Interest Declaration**

More information and definitions can be found here:
https://sporevidencealliance.ca/about/policies-procedures/

Do you have any real, potential, or perceived conflicts of interest related to this work?

- ◯ No
- ◯ Yes, please describe

* 10. **Contact information (email address)**

3. Preventing Falls and Reducing Injury from Falls (4th ed.).,Registered Nurses' Association of Ontario, Editor. 2017, Registered Nurses' Association of Ontario: Toronto.

Economic Impact of Falls Prevention Interventions - Model Conceptualization Survey #1

Thank you for participating in this survey.

Please feel free to contact us with any questions.

Hailey.Saunders@unityhealth.to
Wanrudee.Isaranuwatchai@unityhealth.to

You will receive results of the survey by email in about 2 weeks.

References

1. Eddy, D.M., et al., Model transparency and validation: a report of the ISPOR-SMDM Modeling Good Research Practices Task Force–7. Medical Decision Making, 2012. 32(5): p. 733-743.
2. Siebert, U., et al., State-transition modeling: a report of the ISPOR-SMDM modeling good research practices task force–3. Medical Decision Making, 2012. 32(5): p. 690-700.
3. Preventing Falls and Reducing Injury from Falls (4th ed.).,Registered Nurses' Association of Ontario, Editor. 2017, Registered Nurses' Association of Ontario: Toronto.
