# Supplementary material for: Developing a fall prevention intervention economic model
Source: PLoS One. 2023 Jan 27;18(1):e0280572. doi: 10.1371/journal.pone.0280572 (PMC9882648; doi:10.1371/journal.pone.0280572)
Supplement: S4 File — (PDF) [file pone.0280572.s004.pdf]

Economic Impact of Falls Prevention Interventions - Model Conceptualization Survey #2 [redacted]

INTRODUCTION

Thank you for continuing to participate in our modified Delphi process for the economic impact of falls prevention interventions project.

This process aims to assist in the construction of a model that will simulate patients over their lifetime to estimate the cost-effectiveness of our identified falls prevention interventions through a series of online surveys.

For more information on the overall project please visit the link below containing background documents such as meeting summary #1 and the originally proposed model.
Background documents: [redacted]

OVERALL OBJECTIVE OF THE STUDY (AND MODEL)
We are aiming to create a model to represent the clinical pathway of elderly Canadians (≥ 65 years) at risk of falls and living in a community or residential care setting.

Consequently, we have three specific goals to aid with this process.

- **Goal 1:** to obtain high agreement (≥ 80%) on the set of health states and events to be included in our model.
- **Goal 2:** to obtain high agreement (≥ 80%) on the set of patient attributes associated with falls, costs, and quality of life to be included in our model.
- **Goal 3:** to establish face validity (i.e., whether the structure and pathways of the model accurately reflect the clinical pathways of our population and interventions) of the model structure [1].

As goals 1 and 2 of this process are to obtain high agreement, we may require multiple rounds (maximum being three) to reach these goals.

Goal 3 will be addressed in the next survey.

Note that your opinions will be **anonymous** to others on the panel. The planning committee will send your individual responses back to you along with summary measures of the panel's responses. Therefore, your responses will not be anonymous to the planning committee; however, they will only be shared individually back to you and otherwise circulated as aggregated results.

We anticipate that all sections of this survey will take 15 - 20 minutes for you to complete.

1. Eddy, D.M., et al., Model transparency and validation: a report of the ISPOR-SMDM Modeling Good Research Practices Task Force–7. Medical Decision Making, 2012. 32(5): p. 733-743.

Economic Impact of Falls Prevention Interventions - Model Conceptualization Survey #2 [redacted]

INSTRUCTIONS & BACKGROUND

As there are various existing model structures, currently we are showing all potential health states and events. In other words, they are not mutually exclusive. We would like your input on important health state(s) and/or event(s) to consider so we can conceptualize what our preferred model structure would be like.

Additionally, we may find that some conditions can be both risk factors (patient attributes) and health states or events (results of the progression of disease). For example, when thinking about frailty, polypharmacy and depression were identified as both risk factors and health states associated with frailty [2]. In our case, once we determine what is clinically important for falls, we will design a model structure accordingly.

It may not be possible to incorporate every health state, event, and patient attribute considered to be important at the end of this process for reasons such as lack of data and scope of our project.

Regardless, this conceptualization exercise will allow us to work as a group to prioritize what is important (i.e., should be in our model) and acknowledge our assumptions (e.g., what will be left out) in our final model for economic impact of fall prevention interventions.

In this survey (Survey #2), you will see two types of results from Survey #1.

1. **Items for which there was high agreement to be included - no action required**
2. **Items for which agreement was not obtained and you will be asked to <u>rescore</u> them**

2. Haji Ali Afzali, H., et al., Structuring a conceptual model for cost-effectiveness analysis of frailty interventions. PloS one, 2019. 14(9): p. e0222049.

Economic Impact of Falls Prevention Interventions - Model Conceptualization Survey #2 [redacted]

Health states and/or events that achieved high agreement to be included based on survey #1

High Agreement – Included items	Your score	Mean Score
Hip fracture	[redacted]	4.9
Surgery for hip fracture		4.8
Head injury		4.7
Fall		4.6
Long-term care housing (e.g., nursing home)		4.4
Vertebral fracture		4.4
Hospitalization		4.4
Rehabilitation hospitalization		4.4
Specialized dementia care or memory care in Supportive housing (e.g., retirement home)		4.2

No Action Required

Economic Impact of Falls Prevention Interventions - Model Conceptualization Survey #2[redacted]

* 1. **Potential health states and events**

Health states should be included if they have distinct costs, QALYs from other health states.

Events are shorter than one cycle (in our case, last less than one month), may have associated costs and QALYs and may cause someone to move from one health state to another. When selecting whether something should be a health state or event, consider whether there could be ongoing impacts on costs and QALYs.

Please **re-score** the following health states and events based on the strength of their impact (from very weak to very strong) on costs, QALYs, and progression to other health states specifically for older adults at risk of falls in Table 2 below.

	**No** impact (0)	**Very Weak** impact (1)	**Weak** impact (2)	**Moderate** impact (3)	**Strong** impact (4)	**Very Strong** impact (5)	**Don't Know**
**Independent housing (e.g., own home)**<br>Your score: [redacted]<br>Mean score: 3.7	○	○	○	○	○	○	○
**Independent supported living service in Supportive housing**<br>Your score: [redacted]<br>Mean Score: 3.7	○	○	○	○	○	○	○
**Assisted living in Supportive housing (e.g., retirement home)**<br>Your score: [redacted]<br>Mean score: 3.7	○	○	○	○	○	○	○
**Short-term stay in Supportive housing (e.g., retirement home)**<br>Your score: [redacted]<br>Mean score: 3.5	○	○	○	○	○	○	○
**No fall history**<br>Your score: [redacted]<br>Mean score: 2.9	○	○	○	○	○	○	○
**Post-fall**<br>Your score: [redacted]<br>Mean score: 4.4	○	○	○	○	○	○	○
**Wrist fracture**<br>Your score: [redacted]<br>Mean score: 4.0	○	○	○	○	○	○	○

	**No** impact (0)	**Very Weak** impact (1)	**Weak** impact (2)	**Moderate** impact (3)	**Strong** impact (4)	**Very Strong** impact (5)	**Don't Know**
**Emergency department visit**<br>Your score: [redacted]<br>Mean score: 4.0							
**Fear of falling**<br>Your score: [redacted]<br>Mean score: 3.3							
**Death due to fall**<br>Your score: [redacted]<br>Mean score: 3.7							
**Death**<br>Your score: [redacted]<br>Mean score: 3.5							
**Ankle fracture**<br>*Newly proposed*							
**Humerus fracture**<br>*Newly proposed*							
**Alternate level of care**<br>*Newly proposed*							
**Transitional care unit**<br>*Newly proposed*							

Question **1** of **6**

Economic Impact of Falls Prevention Interventions - Model Conceptualization Survey #2[redacted]

Patient attributes - biological factors that achieved high agreement to be included based on survey #1

High agreement – Included items	Your Score	Mean Score
Gait, balance, or mobility difficulties	[redacted]	5
History of falls/ Previous falls	[redacted]	4.8
Impaired vision	[redacted]	4.5
Age, older age	[redacted]	4.4
Dementia/cognitive impairment	[redacted]	4.4

No Action Required

Economic Impact of Falls Prevention Interventions - Model Conceptualization Survey #2[redacted]

* 2. **Potential patient attributes - biological factors**

Please **re-score** each patient attribute on the strength of its association with falls (from no association (0) to very strong association (5)) including the risk of a fall, risk of injury after a fall, type of injury after a fall, cost of treating an injury after a fall, disease progression, clinical pathway after a fall (e.g., treatment, hospitalization, rehabilitation hospitalization, admittance to long-term care), quality of life, resource use, and mortality. The list of patient attributes are categorized as they are in clinical best practice guidelines, Preventing Falls and Reducing Injury from Falls Fourth Edition [3].

	**No** association (0)	**Very Weak** association (1)	**Weak** association (2)	**Moderate** association (3)	**Strong** association (4)	**Very Strong** association (5)	**Don't Know**
**Malnutrition and related sarcopenia**<br>Your score: [redacted]<br>Mean score: 3.9	○	○	○	○	○	○	○
**Sex**<br>Your score:[redacted]<br>Mean score: 3.4	○	○	○	○	○	○	○
**Incontinence**<br>Your score: [redacted]<br>Mean score: 3.3	○	○	○	○	○	○	○

Question **2** of **6**

Economic Impact of Falls Prevention Interventions - Model Conceptualization Survey #2[redacted]

Patient attributes - behavioural or psychological patient attributes that achieved high agreement to be included based on survey #1

High agreement – Included items	Your Score	Mean Score
Physical inactivity	[redacted]	4.6
Fear of falling	[redacted]	4.2
Substance use	[redacted]	4.1

No Action Required

* 3. **Potential patient attributes - behavioural or psychological factors**

Please **re-score** each patient attribute on the strength of its association with falls (from no association (0) to very strong association (5)) including the risk of a fall, risk of injury after a fall, type of injury after a fall, cost of treating an injury after a fall, disease progression, clinical pathway after a fall (e.g., treatment, hospitalization, rehabilitation hospitalization, admittance to long-term care), quality of life, resource use, and mortality. The list of patient attributes are categorized as they are in clinical best practice guidelines, Preventing Falls and Reducing Injury from Falls Fourth Edition [3].

	**No** association (0)	**Very Weak** association (1)	**Weak** association (2)	**Moderate** association (3)	**Strong** association (4)	**Very Strong** association (5)	**Don't Know**
**Hurrying, not paying attention**<br>Your score: [redacted]<br>Mean score: 3.8	○	○	○	○	○	○	○
**Taking risks**<br>Your score: [redacted]<br>Mean score: 3.6	○	○	○	○	○	○	○
**Dual tasking**<br>Your score:[redacted]<br>Mean score: 3.7	○	○	○	○	○	○	○
**Incorrect use of assistive devices**<br>Your score:[redacted]<br>Mean score: 3.8	○	○	○	○	○	○	○
**Wearing unsupportive footwear**<br>Your score: [redacted]<br>Mean score: 3.7	○	○	○	○	○	○	○
**Gender**<br>Your score: [redacted]<br>Mean score: 2.7	○	○	○	○	○	○	○

Question **3** of **6**

Economic Impact of Falls Prevention Interventions - Model Conceptualization Survey #2[redacted]

Potential patient attributes - environmental or situational factors that achieved high agreement to be included based on survey #1

High agreement – Included items	Your Score	Mean Score
Use of certain medications (anticonvulsants, tranquilizers, antihypertensives, opioids/narcotics)	[redacted]	4.8
Need for transfer assistance	[redacted]	4.4
Home hazards (e.g., loose carpets, pets, stairs)	[redacted]	4.3
Use of restraints	[redacted]	4.2

No Action Required

Economic Impact of Falls Prevention Interventions - Model Conceptualization Survey #2[redacted]

* 4. **Potential patient attributes - environmental or situational factors**

Please **re-score** each patient attribute on the strength of its association with falls (from no association (0) to very strong association (5)) including the risk of a fall, risk of injury after a fall, type of injury after a fall, cost of treating an injury after a fall, disease progression, clinical pathway after a fall (e.g., treatment, hospitalization, rehabilitation hospitalization, admittance to long-term care), quality of life, resource use, and mortality. The list of patient attributes are categorized as they are in clinical best practice guidelines, Preventing Falls and Reducing Injury from Falls Fourth Edition [3].

	**No** association (0)	**Very Weak** association (1)	**Weak** association (2)	**Moderate** association (3)	**Strong** association (4)	**Very Strong** association (5)	**Don't Know**
**Polypharmacy**<br>Your score:[redacted]<br>Mean score: 4.2	○	○	○	○	○	○	○
**Prolonged hospital stay**<br>Your score: [redacted]<br>Mean score: 4.0	○	○	○	○	○	○	○
**Side rails**<br>Your score: [redacted]<br>Mean score: 3.7	○	○	○	○	○	○	○

Question **4** of **6**

Economic Impact of Falls Prevention Interventions - Model Conceptualization Survey #2[redacted]

* 5. **Potential patient attributes - socioeconomic factors**

Please **re-score** each patient attribute on the strength of its association with falls (no association (0) to very strong association (5)) including the risk of a fall, risk of injury after a fall, type of injury after a fall, cost of treating an injury after a fall, disease progression, clinical pathway after a fall (e.g., treatment, hospitalization, rehabilitation hospitalization, admittance to long-term care), quality of life, resource use, and mortality. The list of patient attributes are categorized as they are in clinical best practice guidelines, Preventing Falls and Reducing Injury from Falls Fourth Edition [3].

	**No** association (0)	**Very Weak** association (1)	**Weak** association (2)	**Moderate** association (3)	**Strong** association (4)	**Very Strong** association (5)	**Don't Know**
**Unable to afford supportive footwear**<br>Your score:[redacted]<br>Mean score: 3.8	○	○	○	○	○	○	○
**No social supports, isolated**<br>Your score: [redacted]<br>Mean score: 3.8	○	○	○	○	○	○	○
**Unable to afford certain medications, nutritious foods**<br>Your score:[redacted]<br>Mean score: 3.7	○	○	○	○	○	○	○
**Unable to read**<br>Your score:[redacted]<br>Mean score: 3.0	○	○	○	○	○	○	○

Question **5** of **6**

Economic Impact of Falls Prevention Interventions - Model Conceptualization Survey #2_[redacted]

Potential patient attributes - health conditions that achieved high agreement to be included based on survey #1

High agreement – Included items	Your Score	Mean Score
Overall frailty, older age	[redacted]	4.6
Parkinson's disease	[redacted]	4.6
Stroke	[redacted]	4.6
Dementia/cognitive impairment	[redacted]	4.5
Multiple sclerosis	[redacted]	4.4
Osteoporosis	[redacted]	3.9

No Action Required

Economic Impact of Falls Prevention Interventions - Model Conceptualization Survey #2[redacted]

* 6. **Potential patient attributes - health conditions**

Please **re-score** each patient attribute on the strength of its association with falls (no association (0) to very strong association (5)) including the risk of a fall, risk of injury after a fall, type of injury after a fall, cost of treating an injury after a fall, disease progression, clinical pathway after a fall (e.g., treatment, hospitalization, rehabilitation hospitalization, admittance to long-term care), quality of life, resource use, and mortality. The list of patient attributes are categorized as they are in clinical best practice guidelines, Preventing Falls and Reducing Injury from Falls Fourth Edition [3].

	**No** association (0)	**Very Weak** association (1)	**Weak** association (2)	**Neutral** association (3)	**Strong** association (4)	**Very Strong** association (5)	**Don't Know**
**Psychiatric illness (including depression)**<br>Your score: [redacted]<br>Mean score: 3.7	○	○	○	○	○	○	○
**Osteoarthritis**<br>Your score: [redacted]<br>Mean score: 3.4	○	○	○	○	○	○	○
**Cancer**<br>Your score: [redacted]<br>Mean score: 3.3	○	○	○	○	○	○	○
**Hemophilia**<br>Your score: [redacted]<br>Mean score: 3.0	○	○	○	○	○	○	○
**Diabetes**<br>*Newly proposed*	○	○	○	○	○	○	○
**Cardiac disease**<br>*Newly proposed*	○	○	○	○	○	○	○
**Hypertension**<br>*Newly proposed*	○	○	○	○	○	○	○

Question **6** of **6**

Economic Impact of Falls Prevention Interventions - Model Conceptualization Survey #2[redacted]

Thank you for participating in this survey.

Please feel free to contact us with any questions.

Hailey.Saunders@unityhealth.to
Wanrudee.Isaranuwatchai@unityhealth.to

You will receive results of the survey by email two weeks after the survey closes.

References

1. Eddy, D.M., et al., Model transparency and validation: a report of the ISPOR-SMDM Modeling Good Research Practices Task Force–7. Medical Decision Making, 2012. 32(5): p. 733-743.
2. Haji Ali Afzali, H., et al., Structuring a conceptual model for cost-effectiveness analysis of frailty interventions. PloS one, 2019. 14(9): p. e0222049.
3. Preventing Falls and Reducing Injury from Falls (4th ed.).,Registered Nurses' Association of Ontario, Editor. 2017, Registered Nurses' Association of Ontario: Toronto.
