# Supplementary material for: Developing a fall prevention intervention economic model
Source: PLoS One. 2023 Jan 27;18(1):e0280572. doi: 10.1371/journal.pone.0280572 (PMC9882648; doi:10.1371/journal.pone.0280572)
Supplement: S1 Table — (DOCX) [file pone.0280572.s005.docx]

Supplementary Table 5 Health states of cost-effectiveness state-transition models for fall prevention interventions

| **Reference** | **Population** | **Model type;**  **Time Horizon;**  **Cycle length** | **States** | **Events** |
| --- | --- | --- | --- | --- |
| Church et al. 2011 (22) | People aged 65 and older living in community and residential aged care facilities | Markov model (cohort); 10 years; 1 year | • Low risk faller (individuals who have never fallen) • Medium risk faller (individuals who have previously fallen but incurred no injury) • High risk faller (previously injured individual who fell) • Residential aged care • Death | e.g., Presenting to emergency department, admitted to hospital |
| Church et al. 2012 (23) | People aged 65 and older living in a community setting | Markov model (cohort); lifetime; 1 year | • Low risk faller (individuals who have never fallen) • Medium risk faller (individuals who have previously fallen but incurred no injury) • High risk faller (previously injured individual who fell) • Residential aged care • Death | Fall, emergency attendance, other medical attendance, no injury, discharged to respite RAC, discharged back to community |
| Church et al. 2015 (24) | Older people living in residential aged care facilities | Markov model (cohort); lifetime; 1 year | • Low risk faller (individuals who have never fallen) • Medium risk faller (individuals who have previously fallen but incurred no injury) • High risk faller (previously injured individual who fell) • Death | Fall, emergency attendance, other medical attendance, not injured |
| Deverall et al. 2018 (32)  (Extension of Pega et al. 2016) | Community-dwelling older adults aged ≥65 years | Markov model (cohort); 25 years; 1 year | • High risk group (any previous injurious fall), unmodified house  • High risk group (any previous injurious fall), modified house  • Low risk group (no previous injurious fall), unmodified house  • Low risk group (no previous injurious fall), modified house  • Long-term care  • Die from fall  • Die from other cause | Not reported but could infer injurious fall, and hospitalization |
| Farag et al. 2015 (25) | Community dwelling men and women aged ≥65 years with no prior history of falls | Markov model (cohort); lifetime; NR | • community dweller with no history of falls • community dweller with a prior history of falling • community dweller with a history of prior hospitalization • resident in aged care facility • Death | Fall, treatment |
| FEMOR 2008 (31) | seniors in the community at risk for falling | Markov model (cohort); lifetime; 1 year | • Low risk for falling (no previous medical contact for a fall)  • Moderate risk for falling (1 previous medical contact for a fall that did not result in a fracture)  • High risk for falling faller (2 or more medical contacts for falls and/or any medical contact for fractures)  • Long-term care  • Death | Fall, emergency department, hospital, fracture, non-fracture, rehab |
| Kunigkeit et al. 2018 (33) | patients aged ≥80 years who receive non-institutionalized long-term care | Markov model (cohort); 20 years; 6 months | • Health (non-inst.) • Hip fracture (non-inst.) • Nursing home • Post-fracture (non-inst.) • Re-fracture after admission to nursing home • Death | Not reported |
| Lee et al. 2013 (29) | community dwelling women and men aged 65-80 years | Markov model (cohort); 3 years; 1 month | • Stable • Fall without fear of falling • Fall with fear of falling • Death (Fall event outcomes include: no injury, injurious fall without hospitalization, injurious fall with hospitalization) | Fall without injury, injurious fall without hospitalization, injurious fall with hospitalization |
| Mori et al. 2017* (40) | Women aged ≥65 years, non-Hispanic white | Markov model (microsimulation); lifetime; 1 year | • No fracture • Post vertebral fracture • Post hip fracture • Death | hip, clinical vertebral, wrist, or other osteoporotic fracture |
| Müller et al. 2015* (26) | Men and women aged ≥80 years living in a residential aged care facility | Markov model (cohort); 20 years; 1 year (except first year which was divided into four 3-month cycles) | • Well • Hip fracture • Upper limb fracture without a prior hip fracture in nursing home • Upper limb fracture with a prior hip fracture in nursing home • Post-fracture hip • Death | Fall |
| Pega et al. 2016 (27) | Community dwelling men and women aged ≥65 years | Markov model (cohort); lifetime (age 110); 1 year | • High risk group (previous injurious fall), unmodified house • High risk group (previous injurious fall), modified house • Low risk group (no previous injurious fall), unmodified house • Low risk group (no previous injurious fall), modified house  • Long-term care • Die from fall • Die from other cause | Noninjurious fall, Injurious fall, hospitalization, non-hospital healthcare |
| Poole et al. 2015 (28) | Community dwelling men and women aged ≥60 years | Markov model (cohort); 5 years; 1 year | • Well • Minor fall (fall resulting in accident and emergency attendance but no admission) • Major fall (fall resulting in hospital admission) • Care (no fall in cycle and living in care facility) • Death | Fall, emergency department, hospital admission |
| Wilson et al. 2017 (34) | Community dwelling people aged ≥65 years | Markov model (cohort) (same as Pega et al. 2016); lifetime (110 years old); 1 year | • High risk group (previous injurious fall requiring treatment (in the preceding five  years)), unmodified house  • High risk group (previous injurious fall requiring treatment (in the preceding five  years)), modified house  • Low risk group (no previous treatment for injurious falls), unmodified house  • Low risk group (no previous treatment for injurious falls), modified house  • Long-term care  • Die from fall  • Die from other cause | Not reported |

*Main outcome was fracture prevention
